# Early Postnatal Cardiac Stress Does Not Influence Ventricular Cardiomyocyte Cell-Cycle Withdrawal

**DOI:** 10.3390/jcdd8040038

**Published:** 2021-04-07

**Authors:** Marie Günthel, Karel van Duijvenboden, Jorn Jeremiasse, Maurice J. B. van den Hoff, Vincent M. Christoffels

**Affiliations:** Academic Medical Center, Department of Medical Biology, Amsterdam Cardiovascular Sciences, Amsterdam University Medical Centers, 1100 DD Amsterdam, The Netherlands; m.gunthel@amsterdamumc.nl (M.G.); k.vanduijvenboden@amsterdamumc.nl (K.v.D.); jeremiasse29@gmail.com (J.J.); m.j.vandenhoff@amsterdamumc.nl (M.J.B.v.d.H.)

**Keywords:** neonatal heart, volume overload, congenital heart defect, cardiomyocyte cell-cycle

## Abstract

Congenital heart disease (CHD) is the most common birth defect. After birth, patients with CHD may suffer from cardiac stress resulting from abnormal loading conditions. However, it is not known how this cardiac burden influences postnatal development and adaptation of the ventricles. To study the transcriptional and cell-cycle response of neonatal cardiomyocytes to cardiac stress, we used a genetic mouse model that develops left ventricular volume overload within 2 weeks after birth. The increased volume load caused upregulation of the cardiac stress marker *Nppa* in the left ventricle and interventricular septum as early as 12 days after birth. Transcriptome analysis revealed that cardiac stress induced the expression of cell-cycle genes. This did not influence postnatal cell-cycle withdrawal of cardiomyocytes and other cell types in the ventricles as measured by Ki-67 immunostaining.

## 1. Introduction

Congenital heart disease (CHD) is the most common birth defect, with an incidence of 0.8% of all live births [1]. Right after birth, patients with CHD often suffer from cardiac loading abnormalities such as volume or pressure overload [2]. Whereas small muscular ventricular septal defects may close spontaneously, complex CHDs will require surgical correction [2]. Although nowadays childhood survival is excellent and 90% of children born with CHD reach adulthood, pediatric patients are still at risk of developing heart failure during their first year of life [3,4]. In addition, the majority of adult patients develop cardiac complications such as arrhythmias and heart failure, leading to increased re-hospitalization and the need for re-interventions [5]. Little is known about how cardiomyocytes adapt to cardiac loading abnormalities in CHD patients during early postnatal development of the heart or how this influences the development of cardiac complications such as heart failure.

The transition from fetal to postnatal heart is marked by an increase in oxygen and changed hemodynamics. Cardiomyocytes adapt to these changes by switching from glycolytic to fatty acid metabolism [6]. This metabolic transition is accompanied by a change in cardiomyocyte cell-cycle activity. Before birth, the heart grows by proliferation of small cardiomyocytes. After birth, the proliferation rate drops and the heart grows by hypertrophy of the existing cardiomyocytes [7]. In mice, cardiomyocyte proliferation is completed 7 days after birth [8]. After that, cardiomyocytes will undergo incomplete cell-cycles, leading to polyploidization and multinucleation. Most postnatal DNA synthesis is finished at 14 days after birth [8]. In humans, the final number of cardiomyocytes is reached already 1 month after birth, and afterwards, cardiomyocyte cell-cycle activity mainly accounts for polyploidization [9]. The postnatal metabolic switch and cell-cycle withdrawal of cardiomyocytes are suggested to be highly susceptible to perturbations [10,11]. Therefore, we reasoned that early postnatal cardiac stress could alter cardiomyocyte proliferation and metabolic maturation.

Thus far, there is a lack of animal models investigating adaptation of cardiomyocytes to neonatal cardiac stress due to ventricular volume overload. In this study, we used a mouse model that develops severe cardiac abnormalities early after birth [12]. In *Fstl1^KO/fl^*; *Tie2-Cre* mice, Fstl1 was ablated from the endocardial/endothelial cell-lineage under the control of *Tie2-Cre* [12]. After birth, *Fstl1^KO/fl^; Tie2-Cre* (cKO) mice develop progressive malformation of the mitral valves but do not show echocardiographic abnormalities at postnatal day (P) 7. From P10 onwards, the first cKO pups show valvular insufficiency, causing mitral regurgitation and volume overload of the left ventricle. Malformation of the mitral valves develops progressively, and the left ventricle of cKO mice increases in mass and internal diameter. The first cKO mice start dying at P14. At P21, more than 70% of the mice die from heart failure with preserved ejection fraction [12]. 

We performed RNA-sequencing of PCM-1+ cardiomyocyte nuclei of P12 cKO and healthy control mice to investigate the molecular mechanisms that influence cardiomyocyte development during early ventricular volume overload. Heart-to-body-weight ratios (HW/BW) and expression of the atrial natriuretic peptide (*Nppa*) were used as markers to score for cardiac disease state in these mice. Transcriptional profiles of cardiomyocytes from cKO without cardiac stress and control mice showed almost no differences. cKO mice with induced *Nppa* expression and a significantly higher HW/BW expressed genes involved in cell-cycle regulation. However, PCM-1/Ki-67 double staining showed that the increased expression of cell-cycle genes did not translate into cell-cycle activity of cardiomyocytes and non-cardiomyocytes in cKO mice at P12 and P19. 

## 2. Materials and Methods

### 2.1. Ethical Statement

All animal experiments were performed according to the directive 2010/63/EU of the European Parliament. The protocol was approved by the Animal Experimental Committee of the Amsterdam University Medical Center and was carried out according the guidelines of the Dutch government. The experiments were performed under DAE285. 

### 2.2. Animals and Tissues

The *Fstl1^KO/fl^*; *Tie2-Cre* mice were generated as previously described [12]. Both male and female *Fstl1^KO/fl^*; *Tie2-Cre* mice were used for this study. The control mice used for assessment of cell-cycle activity had the following genotype: *Fstl^WT/fl^*, *Fstl1^KO/fl^*, or *Fstl1^WT/fl^; Tie2Cre.* The control mice used for RNA-seq had the following genotype: *Fstl^WT/fl^*. Mice were sacrificed by CO_2_ and cervical dislocation. Hearts were removed and weighed to assess the heart-weight-to-body-weight ratio. For RNA-sequencing, the experiments hearts were immediately snap frozen in liquid nitrogen. For histological analysis, the hearts were flushed with PBS and fixed in 4% paraformaldehyde overnight. Subsequently, the hearts were transferred to 70% ethanol. The tissues were processed in an automated tissue processor through a graded series of ethanol, embedded in paraffin, and sectioned at 7 μm in 4-chamber view.

### 2.3. Immunohistochemistry

For immunohistochemistry, 1/20 sections were deparaffinized in xylene, rehydrated through a graded series of ethanol, and boiled for 5 min in an unmasking solution (H3300, Vector) using a pressure cooker. Sections were stained with antibodies against PCM-1 (diluted 1:400, HPA023370, Atlas Antibodies, Bromma, Sweden, supplied by Bio Connect, Huissen, The Netherlands) and Ki-67 (diluted 1:200, 556003, BD Biosciences, Vianen, The Netherlands). SYTOX Green was used (diluted 1:40.000, S7020, Invitrogen, supplied by Fisher Scientific, Landsmeer, The Netherlands) as a nuclear stain. The antibodies were visualized with the secondary antibodies Alexa647 (diluted 1:250, A-31571, Thermo Fisher) and Alexa555 (diluted 1:250, A-31572, Thermo Fisher). Sections were mounted in PBS–glycerol (*v*/*v*). 

### 2.4. Quantification of Cardiomyocyte Cell-Cycle Activity

Pictures were taken with a Leica TCS SPE confocal microscope at 40× magnification. For each heart, ca. 3000 total SYTOX Green positive nuclei and ca. 700 PCM-1+ nuclei were scored for co-staining with Ki-67. Double-stained nuclei were manually counted using ImageJ.

### 2.5. In Situ Hybridization

In situ hybridization was performed as described previously [13]. Briefly, sections were deparaffinized and rehydrated to MilliQ. The sections were treated with Proteinase K (25530031, Invitrogen) and were pre-hybridized in a hybridization buffer (50% formamide, 20× SSC, blocking reagent, 0.5 M EDTA, 10% CHAPS, heparine solution, and 10 mg/mL yeast RNA) at 70 °C. Hybridization was performed overnight with a dioxigenin (DIG)-labeled probe against *Nppa* (forward: 5′gggcagagacagcaaacatc 3′, reverse: 5′ cacagtggcaatgtgaccaa 3′). The probes were visualized using alkaline phosphatase-conjugated anti-DIG Fab fragments (11093274910, Roche, Supplier: Merck, Zwijndrecht, The Netherlands) and NBT/BCIP staining reagent (11681451001, Roche, Supplier: Merck, Zwijndrecht, The Netherlands). The sections were dehydrated and mounted in Entellan (107961, Sigma Aldrich, Merck, Zwijndrecht, The Netherlands). Images were acquired with a Leica DM5000 microscope.

### 2.6. Picrosiriusred Staining

For Picrosirius red staining, sections were de-paraffinized in xylene, rehydrated through a graded series of ethanol, and stained for 1 h in Picrosirius red. The sections were washed in acidified water twice for 1 min and dehydrated with a series of graded ethanol up to xylene. The sections were mounted in Entellan (107961, Merck). Images were acquired with a Leica DM5000 microscope.

### 2.7. Cardiomyocyte Nuclei Isolation and Ploidy Analysis

Snap frozen samples of the left ventricle were used for nuclei isolation. Nuclei isolation was performed as described previously [14]. All steps were performed on ice. The samples were homogenized with an Ultra-Turrax homogenizer (IKA, Staufen, Germany) in 3 mL lysis buffer (10 mM Tris-HCl (pH 8.0), 5 mM CaCl_2_, 2 mM EDTA, 0.5 mM EGTA, 1 mM DTT, and 3 mM MgAc). Three milliliters of lysis buffer containing 0.4% triton-X were added, and the suspension was dounced 10 times using a large and small pestle (Wheaton, Vancouver, Canada, supplier: Fisher Scientific, Landsmeer, The Netherlands). The solution was filtered through 100 µm and 30 µm filters (Sysmex, CellTrics, Norderstedt, Germany). The filters were washed with 2 mL lysis buffer containing 0.2% triton-X. The solution was centrifuged at 1000× *g* for 5 min at 4 °C. The pellet was re-suspended in 500 µL staining buffer (2.5% BSA in PBS pH 8.0 and 0.2% Igepal CA-630). The solution was incubated with rabbit anti-PCM-1 (diluted 1:1000, HPA023370, Atlas Antibodies) and Alexa 647 (diluted 1:500, A-31573, Thermo Fisher) for 1 h in total. DAPI was added (0.001 mg/mL, Sigma-Aldrich, D9542, supplier: Merck, Zwijndrecht, The Netherlands) to stain all nuclei. Sorting of the PCM-1 positive nuclei was performed on the BD Influx cell sorter (BD Bioscience, Vianen, The Netherlands). The nuclei were sorted into a lysis buffer of the RNeasy plus micro kit (74034, Quiagen, Venlo, The Netherlands) for subsequent RNA isolation. Ploidy analysis of the sorted nuclei was performed in Flowjo v10.7.1 based on the intensity of DAPI incorporation into on average 20.000 PCM-1 positive nuclei. 

### 2.8. RNA-Sequencing

RNA-sequencing from cardiac nuclei was performed as described previously [14]: 500 pg of RNA was used for cDNA preparation with the Ovation RNA-seq V2 kit (7102-08, TecanNugen, Leek, The Netherlands). Libraries were prepared with the UltraLow V2 kit (0344NB-08, Tecan, Nugen, Leek, The Netherlands), and sequencing was performed on the HiSeq4000 system (Illumnia, San Diego, CA, USA) with 50 bp single-end reads. 

### 2.9. Analysis of RNA-Seq Data

Reads were mapped to the mm10 build of the mouse transcriptome using STAR [15]. Differential expression between groups was determined using the DESeq2 package based on the negative binominal distribution [16]. *p*-values were corrected for multiple testing by using the false discovery rate of Benjamini–Hochberg. Values of *p* < 0.05 were considered statistically significant. Principal component analysis was performed with the DESeq2 package, using default parameters. 

Using DAVID v6.8 (https://david.ncifcrf.gov/ (accessed on 17 December 2020)) gene ontology (GO), term analysis was performed. *p*-values indicated for the GO terms were corrected for multiple testing using Benjamini–Hochberg correction [17,18].

### 2.10. Statistics

Significant differences in the heart to body weights between genotypes were analyzed by unpaired Student’s t tests using Graphpad Prism version 9. *p* < 0.05 was considered significant. 

## 3. Results

### 3.1. Fstl1^KO/fl^; Tie2-Cre Mice Develop Cardiac Stress Soon after Birth

**Figure 1:**
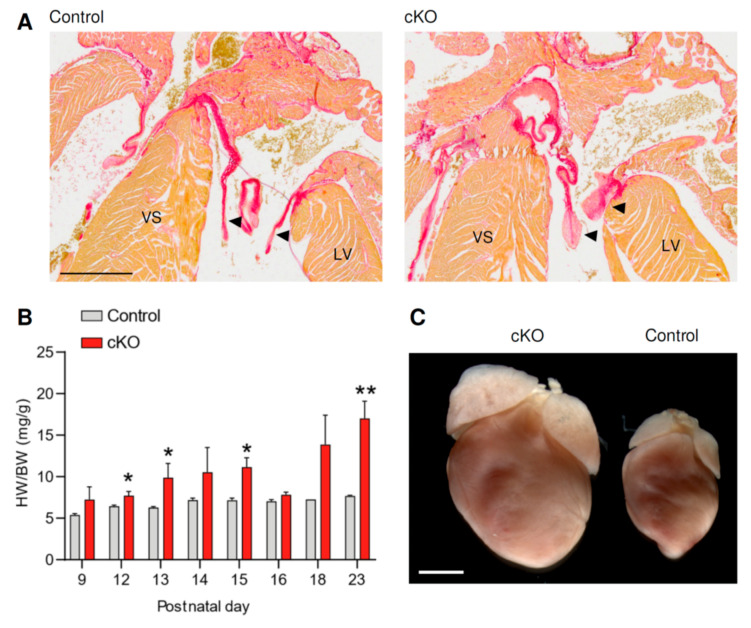
(**A**) Picrosirius red staining shows mitral valves of hearts from control and cKO mice at postnatal day (P) 12. Scale bar indicates 0.5 mm. VS: ventricular septum, LV: left ventricle. Arrowheads indicates mitral valves. (**B**) Hearts of cKO mice at P20 are enlarged. Scale bar indicates 2 mm. (**C**) Heart-weight-to-body-weight ratio (HW/BW) of control and cKO mice at different postnatal stages. * *p* < 0.05, ** *p* < 0.001 (unpaired Student’s *t* test).

We studied *Fstl1^KO/fl^; Tie2-Cre* (cKO) mice to determine the influence of early postnatal cardiac stress on cardiomyocyte gene expression. The phenotype of cKO mice is characterized by the development of malformed mitral valves visible at postnatal day (P) 12 (Figure 1A). The mitral valves become incompetent, causing mitral regurgitation and volume overload of the left ventricle. As a secondary phenotype, the left ventricle of cKO mice dilates and the heart increases in size [12]. At P12, when compared to control (*Fstl^WT/fl^*, *Fstl1^KO/fl^*, or *Fstl1^WT/fl^*; *Tie2Cre*) mice, cKO mice displayed a significant increase in their heart-weight-to-body-weight ratio (HW/BW) due to heart weight increase, as their body-weight did not decrease (Figure 1B, Supplementary Figure S1). At P20, the hearts of cKO mice were visibly enlarged (Figure 1C).

**Figure 2:**
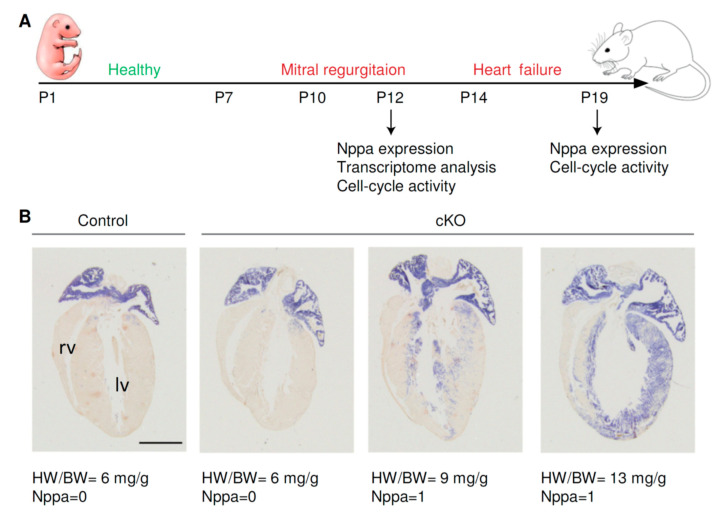
(**A**) Schematic overview of the performed experiments. Starting from postnatal day (P) 10, cKO mice develop mitral regurgitation and heart failure. The transcriptome and *Nppa* expression of cardiomyocytes were analyzed at P12. Cardiomyocyte cell-cycle activity was studied by staining with anti-Ki-67 at P12 and P19. (**B**) In situ hybridization shows the expression of *Nppa* in control and cKO hearts at P12. Mice that express *Nppa* in the left ventricle have higher heart-weight-to-body-weight ratios (HW/BW). According to the expression pattern of *Nppa*, hearts received a *Nppa* score of 0 (*Nppa* not expressed in the left ventricle or only expressed at the base of the in the left ventricle) and *Nppa* = 1 (*Nppa* expression in the left ventricle and luminal side of the interventricular septum). Scale bar indicates 1 mm. lv. Left ventricle; rv, right ventricle.

We used in situ hybridization to assess the expression pattern of *Nppa* in the hearts of cKO and control mice at P12 and P19 (Figure 2A). *Nppa* encodes the hormone atrial natriuretic factor and is broadly used as a cardiac stress marker, induced in ventricles of postnatal hearts undergoing injury or hypertrophy and heart failure [19]. According to the pattern of *Nppa* expression, cardiac stress was scored (Figure 2B, Supplementary Figures S2 and S3). A score of *Nppa* = 0 indicated that *Nppa* expression was comparable to that of control mice. *Nppa* expression in these hearts was restricted to the atria and the base of the heart. Mice with a score of *Nppa* = 1 showed upregulation of *Nppa* in the left ventricle and at the luminar side of the interventricular septum. HW/BW increased from about 6 mg/g in the control hearts and hearts with *Nppa* = 0 to more than 8 mg/g in hearts with a score of *Nppa* = 1. 

### 3.2. Cardiac Stress in Pre-Weaning Mice Does Not Affect Cell-Cycle Activity and Ploidy

**Figure 3:**
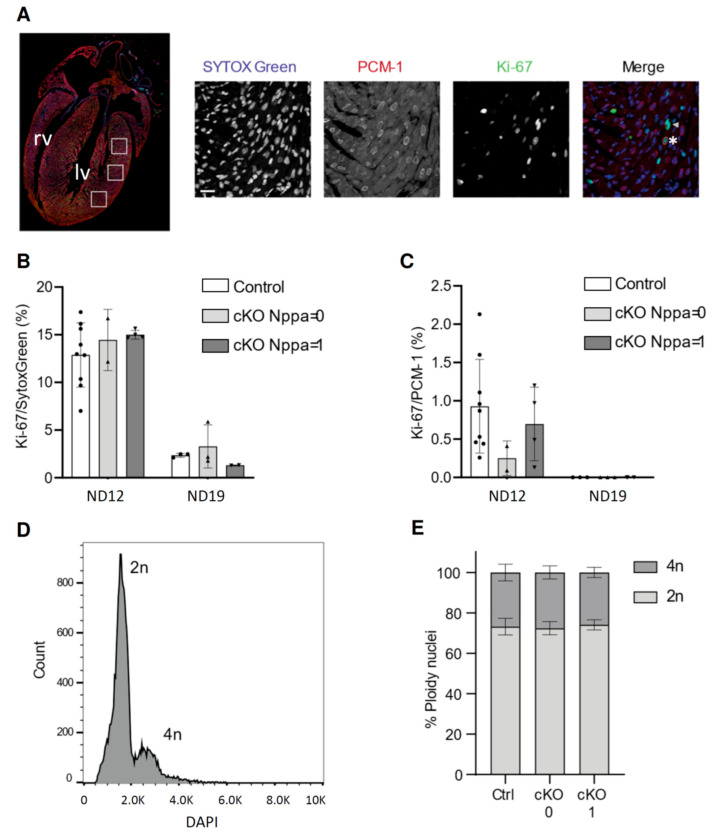
(**A**) Overview of regions within the left ventricle in which cell-cycle activity was determined. (**B**) Immunohistochemical staining of SYTOX Green as nuclear stain, pericentriolar material 1 (PCM-1), and Ki-67 of a part of the left ventricle at postnatal day (P) 12. The arrowhead indicates an example of a Ki-67-positive nucleus. * indicates a Ki-67 and PCM-1 double-positive nucleus. Scale bar indicates 10 μm. (**C**) Ratios of Ki-67-positive nuclei (PCM-1-negative) and Ki-67/PCM-1-positive nuclei in the left ventricles of P12 and P19 mice (P12: N*_Nppa_* = 9, N*_Nppa_*_ = 0_ = 2, N*_Nppa_*_ = 1_ = 4, P19: N_control_ = 3, and N*_Nppa_*_ = 1_ = 4). (**D**) DNA (DAPI intensity) histogram of cardiomyocyte nuclei. (**E**) Percentage of PCM-1-positive nuclei with a diploid (2n) and tetraploid (4n) DNA content of control and cKO mice based on the stress score according their heart-weight-to-body-weight ratios (HW/BW) (N_control_ = 3, N_cKO 0_ = 3, and N_cKO 1_ = 3). Lv. Left ventricle; rv, right ventricle.

To investigate the response of postnatal cell-cycle activity to stress, we determined the cell-cycle activity of cardiomyocytes of the left ventricle of cKO and control mice by performing immunostaining for Ki-67 (Figure 2A and Figure 3A). We chose time points P12, at which the first mice will have started to develop mitral regurgitation and cardiac stress (Figure 2A) and P19. Ki-67 was expressed throughout all phases of the cell-cycle except G0 [20]. Cardiomyocytes that were in cell-cycle were identified by co-staining for PCM-1, specifically staining the nuclear membrane of cardiomyocytes [8]. In addition, we identified all nuclei that were in cell cycle but negative for PCM-1 (mostly endothelial cells and fibroblasts) by co-staining with SYTOX Green. According to their pattern of *Nppa* expression, the samples were given *Nppa* scores of 0 or 1 (Figure 3B and Supplementary Figures S2 and S3). At P12, cell-cycle activity was not significantly altered between cKO (*n* = 4) and control (*n* = 9) samples. The fraction of Ki-67-positive non-cardiomyocyte nuclei varied between 7 and 16%. At P19, only 1–6% of cells of cKO (*n* = 5) and control (*n* = 3) samples were in cell cycle. The percentage of cycling cardiomyocytes was much lower, with on average 0.78% of cardiomyocytes being in cell cycle at P12 (Figure 3B). At P19, we could not identify any cardiomyocyte nuclei that were positively stained for Ki-67 (Figure 3C). Starting around the second postnatal week, cardiomyocytes undergo polyploidization, increasing their DNA content from diploid (2n) to tetraploid (4n) [8]. We therefore assessed the degree of polyploidization of cardiomyocyte nuclei isolated at P12 according to their intensity in the DAPI signal measured during flow cytometry (Figure 3D,E; Supplementary Figure S4). The percentage of diploid and tetraploid cardiomyocyte nuclei was comparable between control mice (*n* = 3), unstressed cKO mice (*n* = 3), and cKO mice with a stress score of 1 (*n* = 3). At P12, approximately 75% of cardiomyocyte nuclei were diploid and 25% were tetraploid (Figure 3E). 

### 3.3. Neonatal Cardiac Stress Induces Cell-Cycle Gene Expression

**Figure 4:**
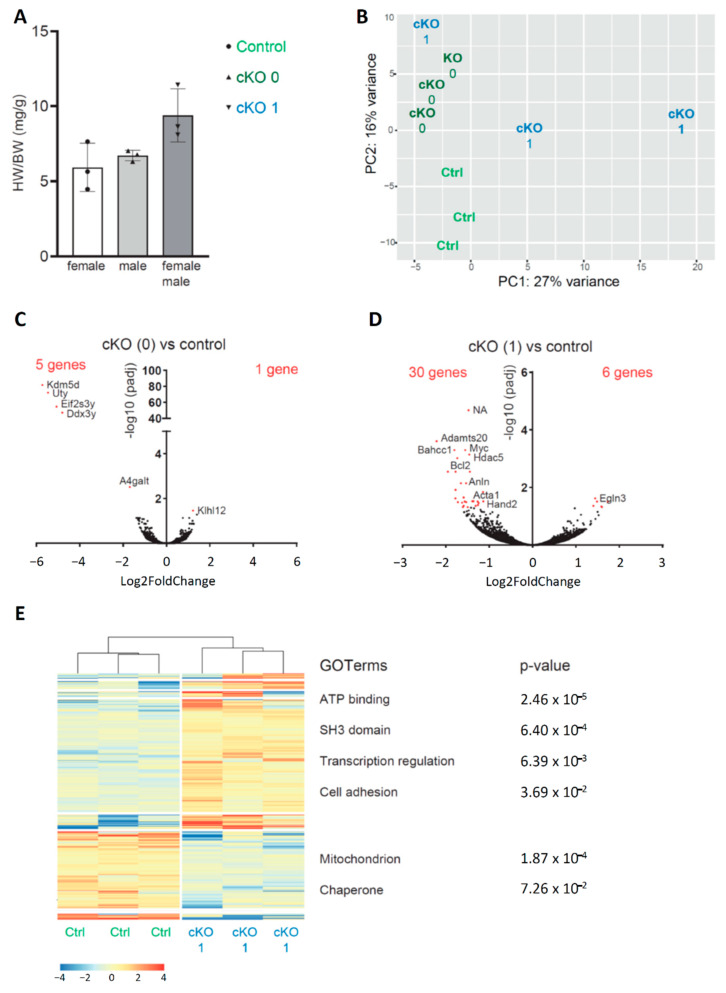
(**A**) Heart-weight-to-body-weight ratio (HW/BW) of mice used for sequencing (control: *n* = 3, cKO: *n* = 5). cKO mice with a HW/BW < 8 mg/g received a stress score of 0. cKO mice with HW/BW > 8 mg/g received a stress score of 1. (**B**) Principal component analysis shows clustering of the control samples and samples with a score of 0. Control samples and samples with different HW/BW scores are marked by different colors. (**C**) Volcano plot shows differential gene expression between control and cKO mice with a score of 0. Significantly differentially expressed genes (*p*-adjusted for multiple testing, false discovery rate < 0.05) are marked in red. (**D**) Volcano plot shows differential gene expression between samples of control and cKO with a score of 1. Significantly differentially expressed genes (*p*-adjusted for multiple testing, false discovery rate < 0.05) are marked in red. (**E**) Functional annotation heat map based on hierarchical clustering on the most differential expressed genes between control and cKO score = 1 (*p*-value < 0.05) and GO terms of groups of differentially expressed genes. The *p*-value of GO terms is corrected for multiple testing (Benjamini).

To study the response of neonatal cardiomyocytes to cardiac stress, we performed RNA-seq on isolated PCM-1-positive cardiomyocyte nuclei from the left ventricle of P12 mice (Figure 2A and Supplementary Figure S4C). Three control (*Fstl^WT/fl^)* mice and five cKO mice were selected for RNA-seq analysis (Figure 4A). The HW/BW of control mice ranged from 4.5 to 7.6 mg/g. The cKO mice were assigned a cardiac stress score dependent on their HW/BW, as we did not investigate the *Nppa* pattern in these hearts. Three cKO mice had a HW/BW of less than 8 mg/g (6.3–7.0 mg/g) and were assumed to be unstressed (score of 0). The remaining three cKO mice displayed a HW/BW > 8 mg/g, ranging from 8.1 to 11.5 mg/g, and were given a cardiac stress score of 1.

Unsupervised principal component (PC) analysis showed grouping of the healthy control samples as well as samples from the unstressed cKO mice (Figure 4B). The first PC discriminated between the different genotypes of mice, whereas the second PC seemed to reflect the stress phenotype. Samples originating from cardiomyocytes of ventricles with a stress score of 1 did not cluster together, showing that gene expression in these samples was more heterogeneous. To study the impact of the genotype on the cardiomyocyte transcriptome in mice without cardiac stress, we compared the gene expression between control mice and the three unstressed cKO mice. We found that only 6 genes were significantly differentially expressed between these two groups (Figure 4C). The 4 most significantly higher expressed genes by cKO mice (*Kdm5d*, *Uty*, *Eif2s3y*, and *Ddx3y*) (−log10 padj > 40) are located on the Y-chromosome, consistent with the sex of the cKO mice (male) versus control mice (female). The lack of further significantly differentially expressed genes indicate that neither the absence of Fstl1 from endocardium-derived cells in unstressed hearts nor the sex difference greatly impact the transcriptome of cardiomyocytes. 

To assess the effect of cardiac stress, we compared the expression profiles between cKO mice with a stress score of 1 and control samples. Thirty genes were significantly higher expressed in the cKO samples (Figure 4D). These include genes involved in the regulation of the cell cycle and apoptosis such as the proto-oncogene *Myc* as well as *Anln* and *Bcl2,* and genes known to be upregulated in response to cardiac hypertrophy such as *Acta1*. cKO samples showed a significant downregulation of the proapoptotic factor *Egln3*. Similar genes were significantly differentially expressed when comparing cKO mice with a stress score of 1 to control mice and unstressed cKO mice (Supplementary Figure S5). cKO mice expressed 16 genes significantly lower than controls and 42 genes significantly higher, including *Myc*, *Anln*, *Bcl2*, and *Cdkn1a*. We performed cluster analysis of the most differentially expressed genes between control samples and cKO samples with a stress score of 1 (Figure 4E). Gene ontology (GO) analysis revealed that genes expressed at lower levels are involved in mitochondrial processes and chaperone activity. Higher expressed genes are involved in transcription regulation, cell organization (cell adhesion and SH3 domain), and transcriptional organization.

## 4. Discussion

In this study, we aimed to assess how cardiac stress of the growing heart after birth influences the transcriptome of cardiomyocytes. This helps us gain a better understanding of cardiomyocyte adaptation in pediatric patients with CHD. Therefore, we used a mouse model for early abnormal cardiac loading conditions and consequent pediatric heart failure. cKO mice develop cardiac stress resulting from left ventricular volume overload caused by mitral regurgitation starting at P10 and heart failure from P14 onwards [12]. Thus far, only a limited number of studies describes the cardiac response to volume overload and its impact on the cardiomyocyte transcriptome and cell-cycle activity in neonatal mice [21]. Studies have mainly focused on understanding cardiomyocyte renewal after injury, such as apical resection [22,23] and myocardial infarction [24,25,26,27]. Both methods cause massive damage and a high degree of cardiomyocyte apoptosis and infiltration of immune cells [28], a condition that may differ from cardiac stress observed in young patients with abnormal loading due to CHD. 

To assess the degree of cardiac stress in cKO mice, we determined the expression pattern of *Nppa*. By using in situ hybridization, we observed the expression of *Nppa* in the left ventricle and luminal site of the interventricular septum in cKO mice at P12, demonstrating that cardiac tissue in these regions was exposed to cardiac stress [14,19]. In cKO mice expressing *Nppa* in the ventricles, the HW/BW increased to more than 8 mg/g. In the healthy control mice and mice without ventricular *Nppa* expression, the HW/BW was about 6 mg/g. This indicates that *Nppa* can be a useful cardiac disease state marker in neonatal mice. 

In order to study the transcriptional response of cardiomyocytes to cardiac stress, we performed RNA-seq of PCM-1-positive nuclei. We selected the hearts of control and cKO mice according to their HW/BW and gave them a stress score of 0 or 1. Interestingly, the transcriptome between control mice and cKO mice with a stress score of 0 essentially did not differ, apart from the expression of Y-chromosome-linked genes highly expressed by the group of male cKO mice. This indicates that the lack of Fstl1 expression in endothelial cells did not affect cardiomyocyte gene expression in mice without cardiac stress. Furthermore, this also suggests that the impact of sex on the transcriptome of cardiomyocytes at this stage is limited. The transcriptional changes between cKO mice that experienced cardiac stress and control mice revealed a higher expression of genes important in the regulation of cell cycle progression and apoptosis such as *Anln*, *Myc*, and *Bcl2*. Anllin (*Anln*) is known to be important for completion of the final part of cytokinesis, and a defect in localization of Anillin at the cleavage furrow was associated with binucleation in cardiomyocytes [29]. The proto-oncogene *Myc* drives proliferation in various tissues and is upregulated in many tumors [30]. However, although Myc was observed to bind its responsive elements in the heart, it fails to initiate proliferation due to the lack of its transcriptional co-factor P-TEFb [31]. Blc2 plays an important role in the regulation of cell survival, and overexpression of Bcl2 was shown to increase the number of cycling cardiomyocytes [32]. We also observed an upregulation of the cyclin-dependent kinase (CDK) inhibitor gene *Cdkn1a*. The negative cell-cycle regulator *Cdkn1a* becomes highly expressed during postnatal development, preventing cardiomyocyte proliferation [33]. Next to cell-cycle genes, genes involved in cardiac hypertrophy such as *Acta1* and *Hdac5* were also expressed higher in cKO mice. The histone deacytelase 5 (HDAC5) is highly expressed in the heart and regulates the transcription of genes by influencing chromatin state, and together with HDAC9, it suppresses hypertrophic growth under cardiac stress [34]. Our results are similar to a study of early pressure overload in neonatal rats [10] where the authors used bulk RNA-sequencing of right ventricular tissue and found that pressure overload caused an upregulation of genes involved in hypertrophy response as well as cell-cycle regulation. In contrast to our results, they also identified large sets of differentially expressed genes involved in cardiomyocyte maturation. We did not detect significantly differential expression of genes involved in metabolic maturation such as oxidative phosphorylation or glycolysis. However, when comparing the most differentially expressed genes between control and cKO mice, lower expressed genes were involved in mitochondrial processes. The differences between the models (pressure overload versus volume overload) and use of whole right ventricular tissue RNA-seq [10] versus cardiomyocyte-specific nuclear RNA-seq (this study) may account for the differences observed. 

Based on the differential expression of cell-cycle genes, we hypothesized that cardiac stress influences the timing of cell-cycle arrest in cKO mice after birth. We used Ki-67 as a marker for cell-cycle activity, as Ki-67 is expressed during all phases of the cell cycle [20]. It should be noted that Ki67 does not allow us to distinguish between proliferation and events of incomplete cell cycles such as multinucleation or polyploidization [35]. Studies in postnatal mice have identified that the final number of cardiomyocytes is reached 11 days after birth [8]. Accordingly, less than 2% of cardiomyocytes showed Ki-67-positive cardiomyocyte nuclei between postnatal day 14 and 16 [8]. At P12, we could detect only a low percentage of cycling cardiomyocytes (on average only 0.78%) and found no differences between control and cKO mice. At weaning, cardiomyocyte cell–cell cycle activity is completed [8,36], which explains why we could not detect any cycling cardiomyocytes in the ventricles of P19 mice. If early postnatal volume overload were to interfere with cardiomyocyte cell-cycle activity, it is likely that the onset of mitral regurgitation in our model occurs too late during postnatal development as mitral valves start being malformed from P10 onward when almost the final number of cardiomyocytes is reached [8,12]. Accordingly, studies using cardiac injury models have shown that the capacity of the heart to react to injury by proliferation is limited to two to three days after birth [24]. In a mouse model of pressure overload by neonatal transverse aortic constriction (TAC), it could be shown that TAC induced at P7 caused myocardial fibrosis and hypertrophy of cardiomyocytes [21]. However, when TAC was performed at P1, the number of cardiomyocytes positive for the mitosis marker phospho-Histone3 increased significantly when compared to sham mice. In addition, we did also not detect changes in ploidy levels between control and cKO mice. An increase in cardiomyocyte ploidy mainly occurs between the second and third weeks after birth, with a peak at postnatal day 14 [8]. Hence, it is possible that the volume overload could influence the cardiomyocyte ploidy levels after P12. Studies in a rat model of neonatal pressure overload demonstrated that polyploidization and multinucleation were increased upon cardiac stress [10].

Our results show that the murine neonatal heart responds to volume overload with only minor changes in the transcriptome of cardiomyocytes. Although the expression of cell-cycle genes was affected in mice experiencing cardiac stress, cardiomyocyte cell-cycle activity was unchanged. It remains challenging to translate this study to the human situation. In CHD patients, abnormal loading conditions usually occur from birth onward and cardiac maturation events such as the transition to cell-cycle arrest may be influenced by gestation time, age at weaning, as well as the intrauterine environment [37]. For example, in humans, the increase in cardiomyocyte ploidy levels persists until the end of the second decade after birth [9], whereas in mice, this process is completed three weeks after birth [8]. However, our study provides insights into the adaption of neonatal cardiomyocytes to cardiac stress and it will be interesting to investigate if similar transcriptional changes occur in pediatric patients with cardiac volume overload and early heart failure.

## Data Availability

The RNA-seq data has been deposited at the Gene Expression Omnibus under the following accession number GSE165134. An Excel file is available of the RNA sequencing data, containing normalized tag counts and differential expression analysis.

## Supplementary Materials

The following are available online at https://www.mdpi.com/article/10.3390/jcdd8040038/s1, Figure S1: Bodyweight (g) of control and cKO mice during postnatal development, Figure S2: *Nppa* expression in control and cKO mice at postnatal day (P) 12, Figure S3: *Nppa* expression in control and cKO mice at postnatal day (P)19, Figure S4: FACS purification of cardiomyocyte nuclei, Figure S5: Volcano plot shows differential gene expression between control and cKO mice with a score of 1, and control and cKO mice with a score of 0.

## Sample Availability

Not available.
