# Supplementary material for: Early Postnatal Cardiac Stress Does Not Influence Ventricular Cardiomyocyte Cell-Cycle Withdrawal
Source: J Cardiovasc Dev Dis. 2021 Apr 7;8(4):38. doi: 10.3390/jcdd8040038 (PMC8068044; doi:10.3390/jcdd8040038)

Supplemental Figure 1

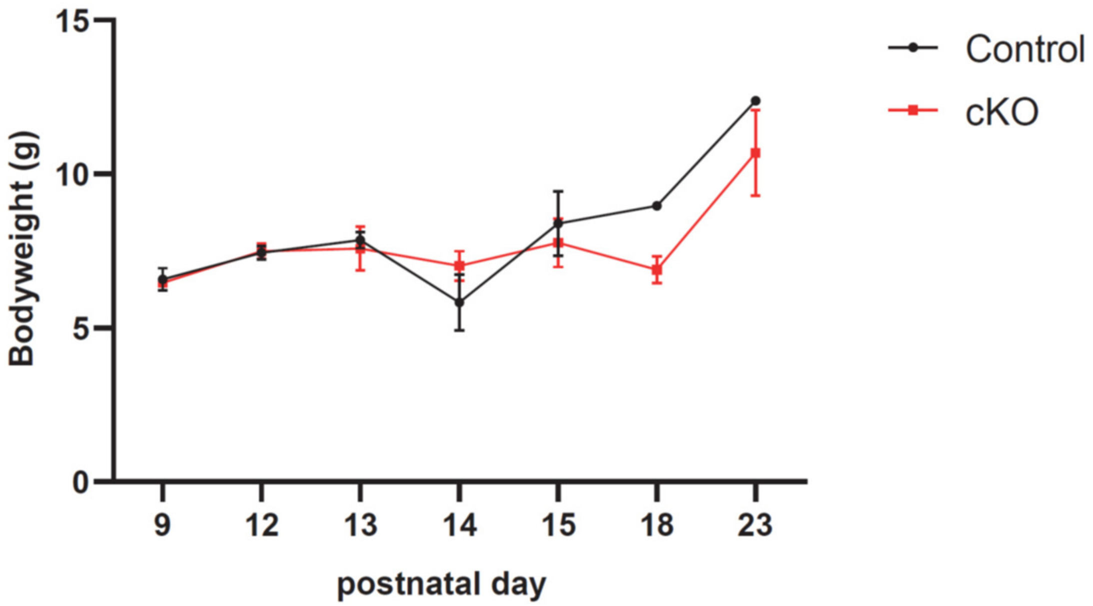

Supplemental Figure 2

Supplemental Figure 2

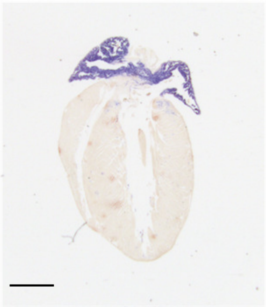

Control
HW/BW=6.2 mg/g
male
Nppa=0

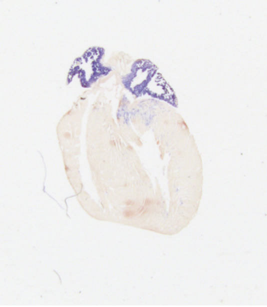

Control
HW/BW=6.9 mg/g
female
Nppa=0

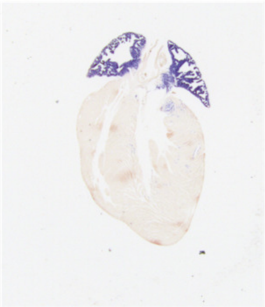

Control
HW/BW=6.6 mg/g
male
Nppa=0

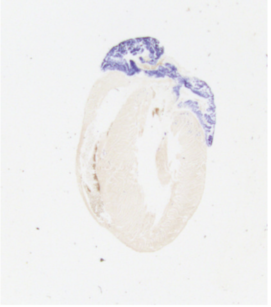

Control
HW/BW=6.2 mg/g
female
Nppa=0

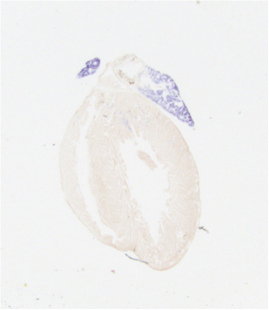

Control
HW/BW=6.1 mg/g
male
Nppa=0

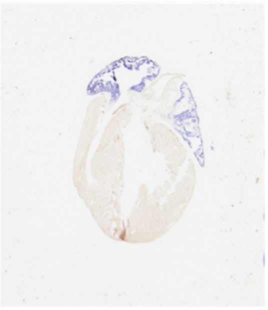

Control
HW/BW=6.0 mg/g
female
Nppa=0

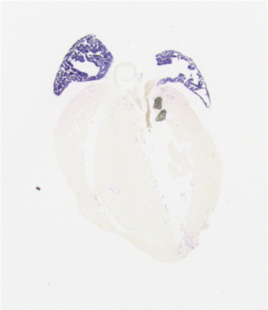

Control
HW/BW=6.1 mg/g
female
Nppa=0

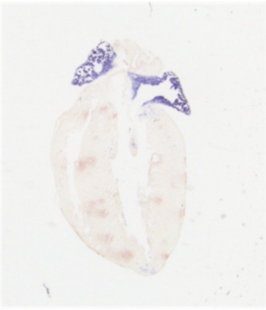

Control
HW/BW=6.3 mg/g
female
Nppa=0

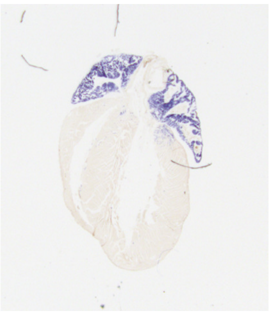

cKO
HW/BW=5.7 mg/g
female
Nppa=0

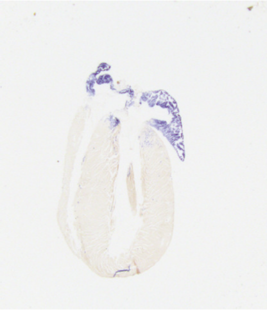

cKO
HW/BW=5.8 mg/g
female
Nppa=0

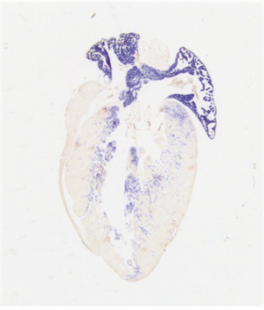

cKO
HW/BW=8.1 mg/g
female
Nppa=1

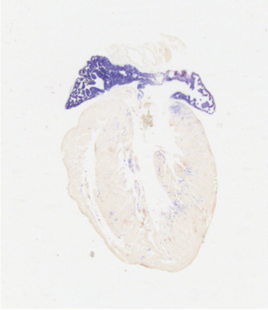

cKO
HW/BW=8.4 mg/g
male
Nppa=1

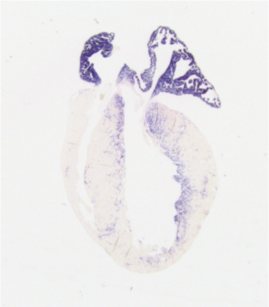

cKO
HW/BW=8.7 mg/g
female
Nppa=1

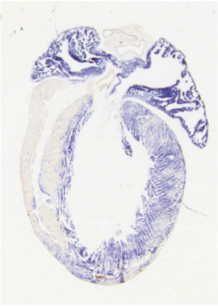

cKO
HW/BW=12.7 mg/g
male
Nppa=1

Supplemental Figure 3

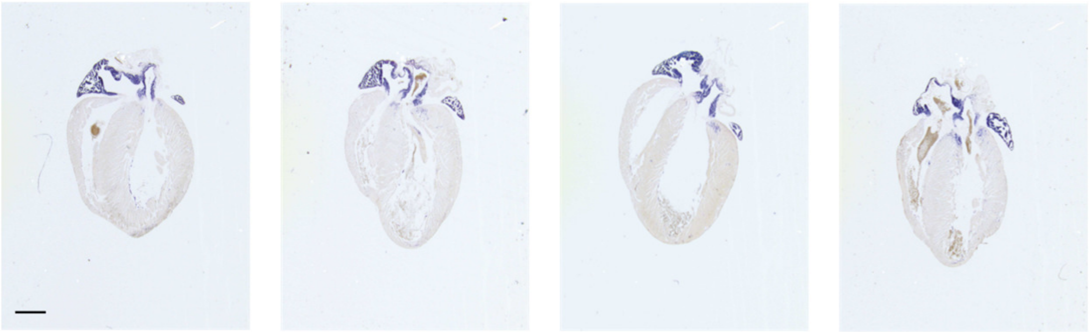

Control
HW/BW=7.3 mg/g
female
Nppa=0

Control
HW/BW=6.8 mg/g
male
Nppa=0

Control
HW/BW=6.1 mg/g
female
Nppa=0

cKO
HW/BW=9.0 mg/g
female
Nppa=0

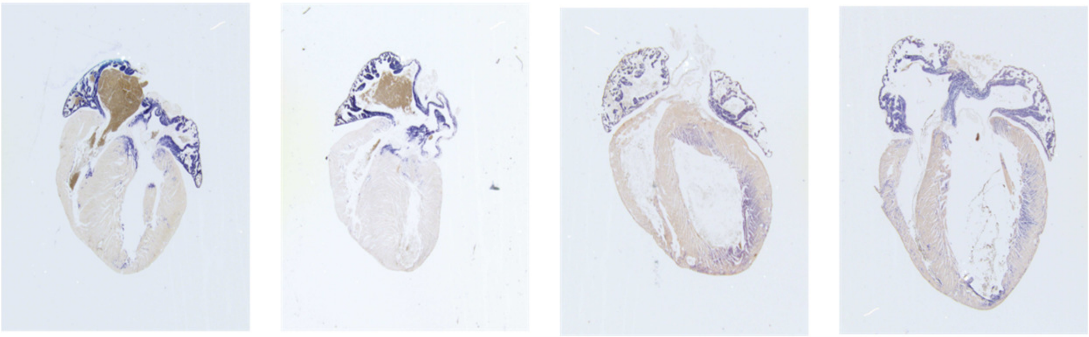

cKO
HW/BW=10.0 mg/g
female
Nppa=0

cKO
HW/BW=9.9 mg/g
female
Nppa=0

cKO
HW/BW=10.6 mg/g
female
Nppa=1

cKO
HW/BW=20.9 mg/g
male
Nppa=1

Supplemental Figure 4

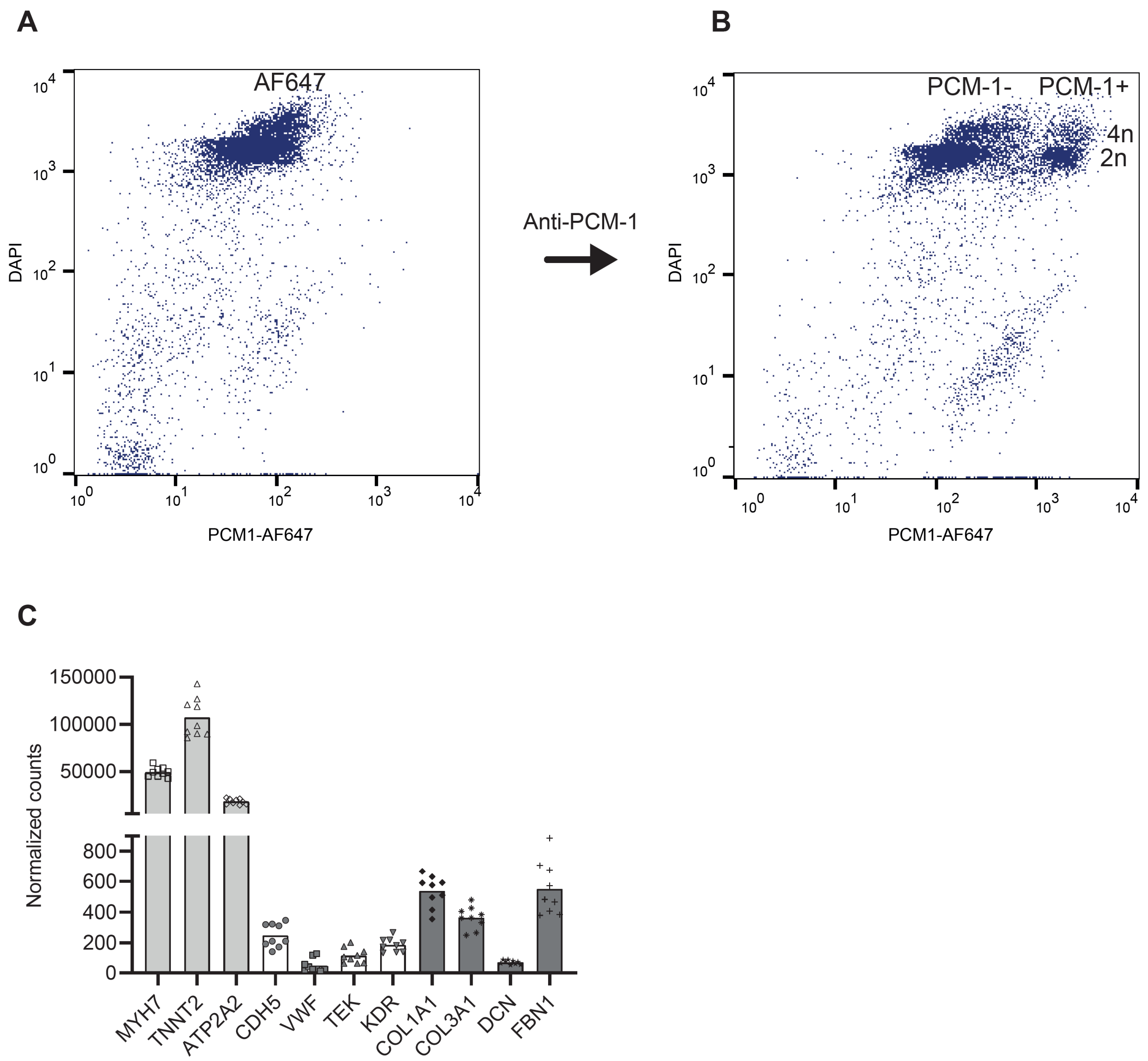

Supplemental Figure 5

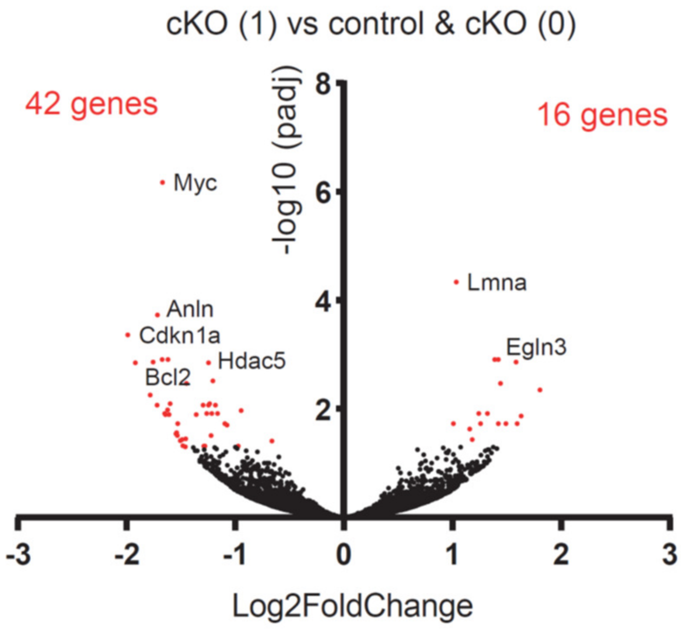

Supplement: Supplementary file 1 [file jcdd-08-00038-s001.pdf]
